# Supplementary material for: Randomized Spatial PCA (RASP): A computationally efficient method for dimensionality reduction of high-resolution spatial transcriptomics data
Source: PLoS Comput Biol. 2025 Dec 10;21(12):e1013759. doi: 10.1371/journal.pcbi.1013759 (PMC12707688; doi:10.1371/journal.pcbi.1013759)
Supplement: Attachment — Submitted filename: rasp_review.pdf [file pcbi.1013759.s002.pdf]

The authors present an interesting method for both cell type clustering and domain identification. Its main novelty lies in the aggregation step using exponentiated inverse distances as aggregation coefficients (at least in the cell type case) and the integration of covariates into the latent embeddings. However, the paper is missing important comparisons with closely related methods such as NichePCA. Also, the hyperparameter tuning strategy seems to favor RASP. If all my comments can be addressed, I think the paper could be a valuable addition to the spatial domain identification method zoo, exploring some new axes of possible modifications to the standard workflow.

Major Comments

- RASP needs to be compared against NichePCA and MENDER.
- It seems the authors tuned the hyperparameters of RASP on each dataset using the ground truth information, while using default parameters for all other methods. If so, a similar hyperparameter tuning procedure would need to be applied to all methods.

Minor Comments

- It would be interesting to see how RASP performs on datasets with larger gene panels, such as Xenium5k and VisiumHD. As far as I am aware, there are no “ground truth” domain annotations available for this data, but a qualitative analysis would still be interesting.
- Expression imputation via spatial smoothing assumes that nearby cells have similar expression levels. However, this is not necessarily true and depends on the tissue. This should be clearly stated.
- The authors should refer to the spatial smoothing matrix as the inverse-distance matrix; otherwise, it can be easily misunderstood as the true inverse.
- The comparison to standard PCA in terms of computational efficiency is problematic, as the sklearn implementation, which the authors are using, already utilizes random PCA at a certain data size.
- Claims about computational efficiency advantages should be toned down, considering NichePCA is as efficient.
- For Figure 3, it should be made clear that they are considering the cell type clustering case. At least in the caption, but even better, also visually.
- It should be clarified in the caption what the gray dots in Fig. 4B and E mean.
- Is RASP with beta = 0 the same as NichePCA (apart from the covariate integration)? This seems to be the default in the domain identification case. If so, it should be mentioned at least in the methods section. Also, it reduces the novelty of the aggregation step, at least for the domain identification task, which should be reflected in the wording, e.g., in the discussion.
- Extended Data Fig. 6 is not referenced in the main text. Even though the results are relevant to evaluating the covariate integration.
- Some more expressive figure titles in the captions would be nice. Currently, it takes time to grasp the main content/point of a figure.

- Badly formatted reference to algorithm 1 in line 446.
- The default KNN ranges and beta values for the cell type and domain annotation task should be clearly specified somewhere. I find conflicting information; sometimes the default range is 5-50, or 60-100 for K.
- It should be stated that the method expects microns as a unit.
- The plots in the first column of Fig. 7c seem to show raw counts, but the legend says normalized expression. A comparison against the actual log-normalized expression would be more meaningful.
- I can't find the supplementary information for the other domain detection methods, as mentioned in line 535.
- It should be clarified that the weights are normalized to sum to one in all cases (mean aggregation in the beta=0 case).
- At best, RASP should be able to handle multiple samples; the easiest strategy would be to apply Harmony after the PCA computation. The Allen Brain Atlas MERFISH dataset might be a good test case. At least you should mention this simple adaptation in the text.
