# Supplementary material for: Randomized Spatial PCA (RASP): A computationally efficient method for dimensionality reduction of high-resolution spatial transcriptomics data
Source: PLoS Comput Biol. 2025 Dec 10;21(12):e1013759. doi: 10.1371/journal.pcbi.1013759 (PMC12707688; doi:10.1371/journal.pcbi.1013759)
Supplement: Attachment — Submitted filename: Response to Reviewers.docx [file pcbi.1013759.s003.docx]

Response to Reviewers Comments for Randomized Spatial PCA (RASP): a computationally efficient method for dimensionality reduction of high-resolution spatial transcriptomics data
PLOS Computational Biology

**Reviewer #1**

**Comment 1
“RASP is a dimensionality reduction method, not a clustering or domain detection method itself. The cell types and spatial regions are obtained from its output spatially smoothed PCs through clustering, and the resulting cluster resolution depends on KNN and β parameters. This is described in the methods and results of the manuscript, but should be made explicit in the Abstract and Introduction to avoid confusion. Currently it reads like this is a method for spatial region detection in abstract.”**

We thank the reviewer for this important clarification. We completely agree that making explicit in both the Abstract and Introduction that RASP is fundamentally a dimensionality reduction method—and that the cell type and spatial region annotations arise through downstream clustering of the spatially smoothed principal components—is critical to avoid any potential confusion.

Accordingly, we have revised both the Abstract and Introduction to clearly emphasize this point, ensuring that readers understand upfront that RASP itself performs dimensionality reduction, while clustering is applied afterward to identify biological groupings.

**Comment 2
“On line 99, BASS is listed as a ‘spatial domain detection’ method. However, BASS performs joint cell type and domain inference. Additionally, in Fig. 4A (mouse brain), where comparisons are made on cell type classification, it would be more appropriate to use the cell-type labels produced by BASS.”**

We that the reviewer for catching this detail. We updated the language describing BASS, and used the cell-type labels produced by BASS in Figure 4A.

**Comment 3
“Gene expression reconstructions are only validated qualitatively. The current manuscript lacks quantitative evaluation on gene expression reconstruction. The authors could consider either predict masked gene expression counts at locations through MSE or correlation, or held-out a subset of genes and quantify reconstruction accuracy.”**

We thank the reviewer for highlighting the importance of quantitative validation for gene expression reconstruction. In this work, rather than performing prediction of masked gene expression or holding out subsets of genes for accuracy assessment, we focused on comparing normalized expression, reconstructed expression, and spatially smoothed reconstructed expression within specifically annotated regions of the dataset.

To quantitatively reflect gene localization, we computed the log2 fold change (log2FC) of the mean expression within the annotated region relative to the background for each of these cases. This approach provides a meaningful metric of improved spatial localization rather than overall predictive accuracy.

We wish to clarify that the gene expression reconstruction feature of RASP is not intended as a gene imputation method aimed at predicting missing genes. Instead, it is primarily designed to enhance the spatial visualization of gene expression patterns by improving localization signals. This distinction underlies our validation strategy and we have now clarified this aspect in the revised manuscript to better communicate the intent and scope of this analysis.

See Section 2 (Results ), Figure 2E, 5E, and and 7E for these changes.

**Comment 4
“The manuscript shows parameter sensitivity analysis and claims that users can tune kNN and β using metrics like Moran’s I and CHAOS. To improve usability, please provide practical defaults and a recommendation table for typical KNN and β values for cell-type annotation vs. spatial domain detection, or preferred clustering algorithms (e.g., Louvain, Leiden), and suggestions for choosing cluster number or resolution in unsupervised settings.”**

We thank the reviewer for this suggestion regarding the provision of practical default parameters and usage recommendations to enhance RASP’s usability. In response, we have included a recommended parameter table within the manuscript that outlines suggested default values for both cell-type annotation and spatial domain detection applications.

Furthermore, in the data analysis figures, we have updated the ARI values reported for RASP to reflect performance obtained using these recommended default parameters, rather than the previous “best-case” scenario where parameters were optimized with knowledge of the true labels. We believe these changes provide clearer and more actionable guidance to users applying RASP in practical scenarios.

See Section 3.1 (Parameter selection considerations), and Table 1 for these changes.

**Reviewer #2 Major Comments**

**Major Comment 1
“RASP needs to be compared against NichePCA and MENDER.”**

We have now included comparisons against both NichePCA and MENDER in the manuscript, and have updated the relevant figures accordingly to reflect these additional benchmarks.

See Section 2 (Results), Figs 2, 4, 5, 6, 7, Extended data figs 6, 9, 12 and 24 for these changes.

**Major Comment 2
“It seems the authors tuned the hyperparameters of RASP on each dataset using the ground truth information, while using default parameters for all other methods. If so, a similar hyperparameter tuning procedure would need to be applied to all methods.”**

We appreciate the reviewer’s comment and agree that fair and comparable parameter tuning is critical. In the revised manuscript, we now present RASP’s performance based on recommended default parameters rather than tuning with access to ground truth, as noted previously.

For other methods, the computational runtime and resource requirements render extensive hyperparameter tuning impractical within a reasonable timeframe. Moreover, several of these methods do not provide explicit guidelines or procedures for systematic parameter optimization.

However, for NichePCA and MENDER — where feasible — we conducted hyperparameter sweeps over key parameters and report the corresponding range of ARI values in the data figures. Details about which parameters were tuned and the ranges explored for these two methods are now included in the Supplemental Information.

See Section 2 (results), Figures 2C, 4B,E, 5C, 6C, 7B, Extended Data Figure 6B, Extended Data Figure 9C, and Extended Data Figure 12C for these changes.

**Minor Comments**

**Minor Comment 1**

**“It would be interesting to see how RASP performs on datasets with larger gene panels, such as Xenium5k and VisiumHD. As far as I am aware, there are no ‘ground truth’ domain annotations available for this data, but a qualitative analysis would still be interesting.”**

We thank the reviewer for this suggestion. We have performed analyses on a VisiumHD dataset and included two additional figures in the Supplement to provide qualitative insights into RASP’s performance on these larger gene panel datasets.

See Extended data figs 24, and 25 for these evaluations

**Minor Comment 2
“Expression imputation via spatial smoothing assumes that nearby cells have similar expression levels. However, this is not necessarily true and depends on the tissue. This should be clearly stated.”**

The reviewer raising important point. To address it, we have added clarifying text in the Discussion to explicitly acknowledge that spatial smoothing assumes spatial expression similarity among neighboring cells, which may not always hold depending on tissue context.

See Section 3.3 (Limitations) for these changes.

**Minor Comment 3
“The authors should refer to the spatial smoothing matrix as the inverse-distance matrix; otherwise, it can be easily misunderstood as the true inverse.”**

We have updated the manuscript accordingly to refer to this matrix as the “inverse-distance matrix” to avoid any potential confusion regarding its interpretation.

**Minor Comment 4
“The comparison to standard PCA in terms of computational efficiency is problematic, as the sklearn implementation, which the authors are using, already utilizes random PCA at a certain data size.”**

Thank you for this clarification. The comparisons we present are indeed against randomized PCA, not standard PCA, in all cases. We have revised the manuscript’s wording to accurately reflect this.

**Minor Comment 5
“Claims about computational efficiency advantages should be toned down, considering NichePCA is as efficient.”**

We have moderated the language surrounding computational efficiency claims accordingly. However, as demonstrated by the runtime comparisons across multiple datasets, RASP continues to outperform NichePCA in many cases.

**Minor Comment 6
“For Figure 3, it should be made clear that they are considering the cell type clustering case. At least in the caption, but even better, also visually.”**

The caption for Figure 3 has been revised to explicitly indicate that the analyses correspond to the cell type clustering case.

See Figure 3 caption for this change.

**Minor Comment 7
“It should be clarified in the caption what the gray dots in Fig. 4B and E mean.”**

Additional details have been added to the caption of Figure 4 to clearly explain the meaning of the gray dots.

**Minor Comment 8
“Is RASP with beta = 0 the same as NichePCA (apart from the covariate integration)? This seems to be the default in the domain identification case. If so, it should be mentioned at least in the methods section. Also, it reduces the novelty of the aggregation step, at least for the domain identification task, which should be reflected in the wording, e.g., in the discussion.”**

While RASP with beta = 0 with shares some conceptual similarity to NichePCA, the methods differ in key implementation details. According to the NichePCA manuscript, their algorithm first aggregates mean expression of every gene across k-nearest neighbors prior to PCA reduction, followed by standard clustering. By contrast, RASP performs spatial smoothing after PCA reduction, which we believe contributes to its improved performance over NichePCA.

Notably, NichePCA’s GitHub repository includes an option to aggregate the PCs instead of the genes, which is akin to RASP’s default domain identification setting with beta=0 . However, the authors do not provide evaluation results or guidelines for this mode. Further, RASP’s tunable parameter provides users with more flexibility to control the degree of spatial smoothing, representing a significant advantage beyond simple averaging of neighboring cells.

We have added these clarifications to the Methods and Discussion sections to better contextualize the relationship and distinctions between RASP and NichePCA.

**Minor Comment 9
“Extended Data Fig. 6 is not referenced in the main text. Even though the results are relevant to evaluating the covariate integration.”**

We thank the reviewer for pointing this out. A reference to Extended Data Figure 6 has now been added to the main text where the covariate integration results are discussed.

See Section 2.4 (Incorporation of covariates improve RASP annotation of mouse ovary ST data), line 169.

**Minor Comment 10
“Some more expressive figure titles in the captions would be nice. Currently, it takes time to grasp the main content/point of a figure.”**

We have revised the figure captions across the manuscript to include clearer, more informative titles and descriptions that better convey the key messages of each figure.

**Minor Comment 11
“Badly formatted reference to algorithm 1 in line 446.”**

The formatting of Algorithm 1 and its reference in the text have been corrected for clarity.

See line 496.

**Minor Comment 12
“The default KNN ranges and beta values for the cell type and domain annotation task should be clearly specified somewhere. I find conflicting information; sometimes the default range is 5-50, or 60-100 for K.”**

We have reviewed the manuscript and updated all references to the default kNN and beta parameter ranges to ensure consistency throughout the text.

**Minor Comment 13
“It should be stated that the method expects microns as a unit.”**

RASP does not require spatial coordinates to be in microns specifically; the method operates on k-nearest neighbor graphs constructed from the coordinate data regardless of unit scale.

**Minor Comment 14
“The plots in the first column of Fig. 7c seem to show raw counts, but the legend says normalized expression. A comparison against the actual log-normalized expression would be more meaningful.”**

We have updated Figure 7c to display normalized expression values consistent with the legend, rather than raw counts, for more meaningful comparison.

**Minor Comment 15
“I can’t find the supplementary information for the other domain detection methods, as mentioned in line 535.”**

We have added the relevant supplementary information detailing the parameter choices and implementation for the other domain detection methods to the supplement.

See SI 1.7 (Implementing other methods) for these changes.

**Minor Comment 16
“It should be clarified that the weights are normalized to sum to one in all cases (mean aggregation in the beta=0 case).”**

A statement clarifying that the weights used in the aggregation step are normalized to sum to one has been added to the algorithm description in the Methods section.

See Algorithm 1, line 7.

**Minor Comment 17
“At best, RASP should be able to handle multiple samples; the easiest strategy would be to apply Harmony after the PCA computation. The Allen Brain Atlas MERFISH dataset might be a good test case. At least you should mention this simple adaptation in the text.”**

We acknowledge the value of this suggested extension. While integration of multiple samples is beyond the scope of the current manuscript, we have added text to the Future Directions section discussing this and noting that applying batch correction methods such as Harmony after PCA would be a straightforward approach.

See Section 3.4 (Future directions) for this language.
